# Influence of Multi-Gene Allele Combinations on Grain Size of Rice and Development of a Regression Equation Model to Predict Grain Parameters

**DOI:** 10.1186/s12284-015-0066-1

**Published:** 2015-10-30

**Authors:** Chan-Mi Lee, Jonghwa Park, Backki Kim, Jeonghwan Seo, Gileung Lee, Su Jang, Hee-Jong Koh

**Affiliations:** Department of Plant Science, Plant Genomics and Breeding Institute, Research Institute for Agriculture and Life Sciences, Seoul National University, Seoul, 151-921 South Korea

**Keywords:** Rice, Grain Size, Regression Equation Model, Allelic Combination

## Abstract

**Background:**

Grain size is one of the key factors determining yield and quality in rice. A large number of genes are involved in the regulation of grain size parameters such as grain length and grain width. Different alleles of these genes have different impacts on the grain size traits under their control. However, the combined influence of multiple alleles of different genes on grain size remains to be investigated. Six key genes known to influence grain size were investigated in this study: *GS3*, *GS5*, *GS6*, *GW2*, *qSW5*/*GW5*, and *GW8*/*OsSPL16.* Allele and grain measurement data were used to develop a regression equation model that can be used for molecular breeding of rice with desired grain characteristics.

**Results:**

A total of 215 diverse rice germplasms, which originated from or were developed in 28 rice-consuming countries, were used in this study. Genotyping analysis demonstrated that a relatively small number of allele combinations were preserved in the diverse population and that these allele combinations were significantly associated with differences in grain size. Furthermore, in several cases, variation at a single gene was sufficient to influence grain size, even when the alleles of other genes remained constant. The data were used to develop a regression equation model for prediction of rice grain size, and this was tested using data from a further 34 germplasms. The model was significantly correlated with three of the four grain size-related traits examined in this study.

**Conclusion:**

Rice grain size is strongly influenced by specific combinations of alleles from six different genes. A regression equation model developed from allele and grain measurement data can be used in rice breeding programs for the development of new rice varieties with desired grain size and shape.

**Electronic supplementary material:**

The online version of this article (doi:10.1186/s12284-015-0066-1) contains supplementary material, which is available to authorized users.

## Background

Rice is one of the most important crops in the world alongside wheat and maize. The top five countries for rice production and consumption are China, India, Indonesia, Bangladesh, and Vietnam. According to a 2009 survey, the combined population of these countries represented nearly half of the total world population (http://data.worldbank.org/). Numerous additional countries are also engaged in rice production and consumption, as well. This indicates the importance of rice as a staple food crop worldwide, and particularly in Asia. The world population continues to increase rapidly and this increase has led to a growing demand for rice. Enhancement of grain yield as well as grain quality is therefore of key agricultural importance.

Rice yield potential is affected by at least four characteristics: panicles per unit area of land, number of spikelets per panicle, percentage of filled grains, and 1000-grain weight (KGW). Of these, KGW is a particularly complex trait that is determined by a combination of grain size-related traits such as grain length (GL), grain width (GW), and grain length to width ratio (LWR) (Tan et al. 2000). Grain size is a further quality trait used by the global market. Grain size preference varies depending on the geographical location. For instance, rice consumers in Thailand, Lao PDR, Cambodia, Malaysia, and the Philippines prefer long and slender grains, while those in Korea, Japan, Northern China and Taiwan prefer shorter and plumper grains (Calingacion et al. 2014). Grain size therefore influences the market available for any given crop (Redoña and Mackill 1998). A combination of different factors influence the grain size of rice. These include GL, GW, LWR, and grain thickness, all of which significantly correlate with grain weight (Tan et al. 2000). In summary, rice grain size and yield are closely related to one another and can be explained by a combination of grain size-related traits.

A large number of genes are associated with rice grain size, and many of their functional roles have been elucidated in a range of studies (Zuo and Li 2014). In most cases, Quantitative trait loci (QTLs) linked to grain size parameters, such as length and width, allowed genes involved in grain size regulation to be fine mapped and characterized. For instance, *GRAIN WIDTH and WEIGHT2* (*GW2*) gene, which encodes a RING-type E3 ubiquitin ligase, was fine mapped from a major QTL responsible for rice grain width and weight (Song et al. 2007). From two rice varieties showing contrast grain width, Fengaizhan 1 (FAZ1; small-grain *indica*-type variety) and Wuyujing 3 (WY3; large-grain *japonica*-type variety), two alleles of *GW2* gene were distinguished by a single nucleotide polymorphism (SNP) in exon 4. The SNP resulted in a premature translational termination of GW2 protein, and was thought to be responsible for the enhanced grain width and weight in WY3 compared to FAZ1. *GRAIN SIZE 3* (*GS3*) gene, which encodes a putative transmembrane protein, was fine mapped from a major QTL responsible for grain length and weight (Fan et al. 2006). The functional role of GS3 has been suggested as a negative regulator to prevent the growth of the grain size. Two alleles of *GS3* gene, A and C, were distinguished by a SNP in exon 2 (Takano-Kai et al. 2009). Similarly, this SNP also resulted in a premature translational termination of GS3 protein. It was reported that the allelic variation of *GS3* gene was significantly associated with the different grain length (Fan et al. 2009). In addition, an association study revealed that the A-allele (C165A mutation) of *GS3* gene was significantly linked to enhanced rice grain length (Takano-Kai et al. 2009). *GRAIN SIZE 5* (*GS5*) gene, which encodes a putative serine carboxypeptidase, was fine mapped from a QTL responsible for grain size (Li et al. 2011). It was also suggested that higher expression of *GS5* was significantly correlated with larger grain size. Three alleles of *GS5* gene were distinguished by natural variations in the promoter region, and the rice varieties carrying these alleles also showed significant differences in grain width: H94- (narrow grain), Zhenshan97- (medium grain), and Zhonghua11-allele (wide grain). Therefore, these natural variations in the promoter region were thought to be responsible for the different grain width. *qSW5/GW5* gene was fine mapped from a major QTL responsible for grain width, *QTL for rice seed width on chromosome 5* (*qSW5*) (Shomura et al. 2008). Compared to the Kasalath (*aus*-type variety), the *qSW5/GW5* gene region in Nipponbare (*japonica*-type variety) and in several *indica*-type varieties contained a large deletion and number of SNPs. Three alleles of *qSW5/GW5* gene were distinguished by length of deletion at the *qSW5/GW5* locus (Yan et al. 2011): Kasalath-allele (no deletion), *indica* II-allele (950 bp deletion), and Nipponbare-allele (1,212 bp deletion). Analysis of variance (ANOVA) showed that germplasms carrying the Nipponbare-allele had wider grains than germplasms with the other two alleles (Shomura et al. 2008). *GRAIN WIDTH 8/SQUAMOSA PROMOTER BINDING PROTEIN-LIKE 16* (*GW8*/*OsSPL16*) gene, which encodes a positive regulator of cell proliferation, was fine mapped from a major QTL responsible for grain width, *qGW8* (Wang et al. 2012). It was also demonstrated that higher expression of *GW8*/*OsSPL16* resulted in enhanced rice grain width and yield due to promoted cell division and grain filling. In Basmati 385 (*indica*-type traditional Basmati variety), 10-bp deletion was found in the *GW8*/*OsSPL16* promoter region compared to other two *indica*-type varieties (HJX 74 and TN1), and named Basmati-allele. This deletion was thought to be responsible for the slender grain in Basmati 385 compared to HJX 74. In addition, two alleles of *GW8*/*OsSPL16* gene were also distinguished by a SNP in exon 1: HJX 74- and TN1-alleles. Beside these, several genes involved in regulation of rice grain size were also identified by other approaches. For instance, *GRAIN SIZE 6* (*GS6*) gene was isolated from an ethylmethanesulfonate (EMS)-induced mutant, *gs6* (Sun et al. 2013). *GS6* encodes a member of GRAS family proteins, which plays an important role in reducing rice grain size. Three alleles of the *GS6* gene, type I to III, were distinguished by nucleotide variants in the promoter region. Of these, the type I-allele is found predominantly in *japonica*-type varieties, which have wider and heavier grains than varieties with the other two alleles.

Despite recent advances in our understanding of grain size regulation in rice, the influence of combinations of alleles from multiple different genes remains largely unknown. It is therefore important to understand the influence of allele combinations on grain size when considering breeding for preferred grain shape and subsequently enhanced rice grain yield.

In this study, we examined allele combinations of above-mentioned six key genes that were previously shown to influence grain size. A total of 215 rice germplasms from diverse countries were examined. The data were used to develop a regression equation model with potential for use in molecular breeding of rice. These results will facilitate prediction of grain size and facilitate efficient breeding of rice varieties with desirable grain yield and shape characteristics.

## Results

### Diverse Grain Size in the Rice Germplasm Collection

A total of 215 rice germplasms collected from 28 rice-consuming countries were used to investigate the influence of allele combinations of six key genes on rice grain size (Additional file 1). This collection contained diverse rice germplasms from wild species to modern cultivars: *tropical japonica* (24), *temperate japonica* (63), *indica* (85), *aromatic* (9), *aus* (15), wild species (1), traditional (1), and ungrouped (17). The average grain size differed substantially between the different germplasms with respect to four grain size-related traits (GL, GW, LWR, and KGW). The GL range was 6.09–10.81 mm, GW range was 2.21–4.32 mm, LWR range was 1.79–4.34, and KGW range was 14.49–51.91 g (Additional file 1). Significant correlations between these four traits were observed in the germplasm collection (Additional file 2). This suggests that the four traits determining grain size are tightly related to one another and play important roles in regulating rice grain size.

### Allele Distributions of Six Genes Involved in Determining Grain Size

Numerous genes are associated with rice grain size, and the functional roles of many of their protein products have been elucidated in a range of studies (Zuo and Li 2014). However, the allelic contributions of multiple genes to grain size and shape have not yet been explored. In this study, allelic variation in several genes was used to develop a regression equation model that could be used for molecular breeding in rice. Six key genes were investigated: *GS3* (Os03g0407400), *GS5* (Os05g0158500), *GS6* (Os06g0127800), *GW2* (Os02g0244100), *GW8*/*OsSPL16* (Os08g0531600), and *qSW5*/*GW5* (Os05g0187500). The genes each had two or three functional alleles in the diverse germplasm collection, and grain size parameters such as length and width varied considerably with these alleles. The allele distributions of the six genes were determined in the germplasm collection, then considered in the context of grain measurements.

Two alleles of *GS3* gene, A and C, were distinguished by a SNP in exon 2 (Takano-Kai et al. 2009). A functional nucleotide polymorphism (FNP) marker, GS3-*Pst*I, which was developed by Yan et al. (2011) was used to determine the allele distribution of *GS3* gene in the germplasm collection. A- and C-alleles were found in 27 and 83 germplasms, respectively. Interestingly, an additional allele was also uncovered (named the B-allele) that contained a 45-base pair insertion in the first intron. The B-allele was found in the remainder of the collected germplasms. The additional B-allele necessitated use of a higher percentage agarose gel (2.5 %) than was used previously. The different *GS3* allelic groups exhibited differed grain parameters: (1) germplasms with the A-allele had significantly higher GL and LWR values, and lower GW values, than the other allelic groups; (2) germplasms with the B-allele had intermediate GL and LWR values; and (3) germplasms with the C-allele had significantly lower GL and LWR values, and significantly higher GW values, than the other germplasms (Table 1). In summary, the three alleles of *GS3* gene were significantly associated with differences in grain size-related traits (except for KGW) in the diverse germplasms used in this study.

**Table 1:** Allelic variation of genes involved in regulation of rice grain size

			Grain length (mm)		Grain width (mm)		Grain length to width ratio		1000 grain weight (g)	
	Alleles	Germplasms	Means±SD	*P* value	Means±SD	*P* value	Means±SD	*P* value	Means±SD	*P* value
*GS3*	A-allele	27	9.35±0.49^a^	<.0001*	2.90±0.57^b^	<.0001*	3.35±0.67^a^	<.0001*	28.80±6.83^a^	0.1587
	B-allele	105	8.52±0.93^b^		2.97±0.37^b^		2.92±0.54^b^		26.86±5.40^ab^	
	C-allele	83	7.72±0.86^c^		3.28±0.39^a^		2.41±0.54^c^		26.66±4.12^b^	
*GS5*	Zhenshan97	17	8.65±0.79^a^	0.0145*	2.87±0.34^a^	0.0001*	3.09±0.61^a^	0.0003*	26.40±2.57^a^	0.1293
	H94	63	8.55±0.86^ab^		2.94±0.41^b^		2.98±0.58^a^		26.03±4.00^a^	
	Zhonghua11	135	8.16±1.08^b^		3.18±0.43^b^		2.64±0.64^b^		27.57±5.81^a^	
*GS6*	Type I	101	8.05±1.06^b^	0.0003*	3.30±0.39^a^	<.0001*	2.49±0.55^b^	<.0001*	28.37±5.49^a^	0.0003*
	Type II/III	114	8.55±0.92^a^		2.89±0.38^b^		3.03±0.62^a^		25.84±4.59^b^	
*GW2*	WY3	3	8.91±0.94^a^	0.3093	3.84±0.21^a^	0.0021*	2.31±0.17^a^	0.21	48.54±2.93^a^	<.0001*
	FAZ1	212	8.31±1.02^a^		3.07±0.43^b^		2.78±0.65^a^		26.72±4.52^b^	
*qSW5/ GW5*	Kasalath	90	8.81±0.92^a^	<.0001*	2.77±0.33^c^	<.0001*	3.25±0.58^a^	<.0001*	26.00±4.67^b^	0.0097*
	Indica II	41	8.28±0.75^b^		3.11±0.29^b^		2.68±0.32^b^		26.61±3.51^ab^	
	Nipponbare	84	7.80±0.97^c^		3.41±0.33^a^		2.32±0.46^c^		28.34±6.07^a^	
*GW8/ OsSPL16*	Basmati	111	7.99±1.05^b^	<.0001*	3.27±0.41^a^	<.0001*	2.51±0.61^b^	<.0001*	27.74±5.47^a^	0.0051*
	HJX74	66	8.75±0.81^a^		2.93±0.39^b^		3.06±0.56^a^		27.21±4.65^a^	
	TN1	38	8.51±0.90^a^		2.82±0.32^b^		3.07±0.57^a^		24.62±4.56^b^	

Data represent mean±standard deviation; ANOVA test, *P < 0.05

^a, b,^ and ^c^ were ranked by *Duncan's* test

Three alleles of *GS5* gene, Zhenshan97, H94, and Zhonghua11, were distinguished by natural variations in the promoter region (Li et al. 2011). Two derived cleaved amplified polymorphic sequence (dCAPS) markers were used to investigate the allelic distribution of *GS5* gene in the germplasm collection: (1) GS5-*Taq*I for classification of the H94- and Zhenshan97-alleles, and (2) GS5-*Sal*I to distinguish the Zhonghua11-allele. The Zhonghua11-allele was most abundant (135 out of 215 germplasms) and the Zhenshan97- and H94-alleles were found in 17 and 63 germplasms, respectively. Consistent with the previously published results (Li et al. 2011), the 135 germplasms carrying the Zhonghua11-allele had significantly higher GW values than the other germplasms. In addition to the GW, we also observed significant differences between the allelic germplasm groups for GL and LWR (Table 1).

Three alleles of *GS6* gene, type I to III, were distinguished by nucleotide variants in the promoter region (Sun et al. 2013). For this study, as only the type I-allele was associated with enhanced grain parameters, the type II- and type III-alleles were considered together (i.e., two categories were used: type I, and type II/III). An insertion-deletion (InDel) polymorphism marker, indel-*GS6*, was designed in the promoter region of *GS6* gene to distinguish the different alleles. Type I and II/III-alleles were found in 104 and 114 germplasms, respectively. As noted previously (Sun et al. 2013), germplasms carrying the type I-allele had significantly higher GW and KGW values than the other germplasms (Table 1). In addition to GW and KGW, we also observed significant differences between the allelic germplasm groups for GL and LWR.

Two alleles of *GW2* gene, FAZ1 and WY3, were distinguished by a SNP in exon 4 (Song et al. 2007). A dCAPS marker, GW2-*Sca*I, was designed to examine allele distribution. The FAZ1-allele was found in almost all the germplasms, with only three carrying the WY3-allele (Hokuriku 130, Dearybbyeo 1, and Oochikara). As noted previously (Song et al. 2007), the WY3-allele germplasms had significantly higher GW and KGW values (Table 1). This suggests that the WY3-allele of *GW2* gene is valuable for grain size enhancement, but is relatively rare.

Three alleles of *qSW5/GW5* gene, Kasalath, *indica* II, and Nipponbare, were distinguished by length of deletion at the *qSW5/GW5* locus (Yan et al. 2011). A previously designed InDel marker, N1212del (Shomura et al. 2008), was used to investigate the allele distribution of *qSW5/GW5* gene in the germplasm collection. All three *qSW5/GW5* alleles were found in the collection. The Kasalath-allele was found in 90 germplasms, Indica II-allele in 41 germplasms, and Nipponbare-allele in 84 germplasms. Consistent with the previous study (Shomura et al. 2008), the 84 germplasms carrying the Nipponbare-allele of *qSW5/GW5* gene had significantly higher GW values than the germplasms with the other two alleles (Table 1). In addition, we also observed significant differences between the allelic germplasm groups for GL, LWR, and KGW.

Three alleles of *GW8*/*OsSPL16* gene, Basmati, HJX74, and TN1, were reported previously (Wang et al. 2012). Here, an InDel marker, indel-*GW8*, was designed to identify germplasms carrying the Basmati-allele. In addition, a specific genomic region containing the 5′ untranslated region (UTR) and first exon was sequenced to distinguish between the HJX74- and TN1-alleles. The Basmati-allele of *GW8*/*OsSPL16* was found in 111 germplasms, and 66 and 38 germplasms contained the HJX74- and TN1-alleles, respectively. In contrast with the previous study (Wang et al. 2012), germplasms with the Basmati-allele had wider grains than germplasms carrying the other two alleles. The three alleles of *GW8*/*OsSPL16* gene were significantly associated with differences in GL, GW, LWR, and KGW (Table 1).

Taken together, our data show that allelic variations of the six genes are widely distributed in our germplasm collection. In most cases, allelic variants are significantly associated with differences in three or more of the major traits (GL, GW, LWR, and KGW) for grain size.

### Influence of Allele Combinations on Grain Size-related Traits

The genotyping results showed that several germplasms had a similar grain size and also shared a particular allele combination of the six key genes examined in this study. For example, 33 germplasms had a GL of 6.16–8.15 mm and had the same allele combination (B-allele of *GS3*, Zhonghua11-allele of *GS5*, Nipponbare-allele of *qSW5/GW5*, type I-allele of *GS6*, and Basmati-allele of *GW8*/*OsSPL16*). This indicated that similarity in rice GL could be attributed to certain allele combinations even in the presence of different genetic backgrounds. We therefore grouped our germplasm collection according to allele combinations that were significantly associated with four major traits (GL, GW, LWR, and KGW) for grain size and yield (Additional file 3).

With respect to GL, significant differences were observed between alleles of five genes (*GS3*, *GS5*, *qSW5/GW5*, *GS6*, and *GW8*/*OsSPL16*) (Table 1). Twenty-six of the 162 possible allele combinations for these five genes were found in 166 of the 215 germplasms. These combinations were termed group LA to LZ. Three or more germplasms constituted a group. Several combination groups showed a single gene-specific allelic variation. For instance, group LA, LB, and LC all had the same allele combinations for four genes (*GS5*, *qSW5/GW5*, *GS6*, and *GW8*/*OsSPL16)*, but only differed at *GS3*. Other groups (LD–LF and LJ–LL) also showed single gene-specific allelic variation at *GS3*. Despite the similar allele combination patterns in these groups, average GL was significantly different only for group LA–LC (Additional file 4). These results indicate that allelic variation at *GS3* plays an important role in regulation of rice GL in the presence of a certain allele combination of four other genes (Zhonghua11-allele of *GS5*, Nipponbare-type of *qSW5/GW5*, type I-allele of *GS6*, and Basmati-allele of *GW8*/*OsSPL16*). Although several other groups also varied at only one gene (*GS5*, *qSW5/GW5*, *GS6*, or *GW8*/*OsSPL16*), the average GL was not significantly different between these groups.

As for GW, significant differences were observed between alleles of all six genes (*GW2*, *GS3*, *GS5*, *qSW5/GW5*, *GS6*, and *GW8*/*OsSPL16*) (Table 1). Of the 324 possible allele combinations, 26 were present in a total of 164 germplasms and were named group WA–WZ (Additional file 3). Of these, average GW was significantly different in groups containing single gene-specific allelic variation at one of three genes (*GS3*, *GS6*, or *qSW5/GW5*), particularly when certain allele combinations of the other five genes were present (Table 2). For instance, average GW was significantly different between WN and WV, which different only at *GS6* gene.

**Table 2:** Allele combinations including a single gene-specific allelic variation

Grain size-related traits	Gene name	Group name	Germplasms	*GS3*	*GS5*	*GS6*	*GW2*	*qSW5/*	*GW8/*	*P* value
								*GW5*	*OsSPL16*	
Grain length	*GS3*	LA	9	B	G	N	-	N	B	<0.0001**
		LB	5	A	G	N	-	N	B	
		LC	33	C	G	N	-	N	B	
Grain width	*GS3*	WE	3	A	G	N	F	K	B	0.0324*
		WD	10	B	G	N	F	K	B	
		WG	6	C	G	N	F	K	B	
	*GS6*	WN	3	B	G	N	F	I	T	0.0442*
		WV	3	B	G	O	F	I	T	
	*qSW5/GW5*	WU	12	B	H	O	F	K	J	<0.0001**
		WW	13	B	H	O	F	I	J	
		WY	3	B	S	O	F	K	J	0.0265*
		WZ	3	B	S	O	F	I	J	
		WC	32	C	G	N	F	N	B	<0.0001**
		WI	6	C	G	N	F	K	B	
		WH	10	C	G	O	F	N	B	0.0129*
		WL	6	C	G	O	F	K	B	
Grain length to width ratio	*GS3*	RD	6	A	-	N	-	N	B	
		RA	12	B	-	N	-	N	B	<0.0001**
		RB	37	C	-	N	-	N	B	
	*qSW5/GW5*	RP	20	B	-	O	-	I	J	
		RN	17	B	-	O	-	K	J	<0.0001**
		RJ	3	B	-	O	-	N	J	
		RP	20	B	^-^	O	^-^	I	J	<0.0001**
		RN	17	B		O		K	J	
		RQ	6	B	^-^	O	^-^	I	T	0.0184*
		RO	10	B		O		K	T	
		RE	7	C	^-^	N	^-^	K	B	<0.0001**
		RB	37	C		N		N	B	
1000-grain weight	*GW8/OsSPL16*	KA	52	^-^	^-^	N	F	N	B	0.0276*
		KB	8			N	F	N	J	
		KM	21	^-^	^-^	O	F	I	J	0.0035**
		KN	9			O	F	I	T	

The significance of allelic variation of each gene in corresponding grain size-related trait was indicated by *P* value. ** and * indicate the significance at 1 % and 5 % level, respectively. The following characters represent each allele

A: A-allele type of *GS3*, B: B-allele type of *GS3*, and C: C-allele type of *GS3*

G: Zhonghua11 type of *GS5*, H: H94 type of *GS5*, and S: Zhenshan97 type of *GS5*

N: Type I of *GS6* and O: Type II/III of *GS6*

F: FAZ I type of *GW2*

K: Kasalath type of *qSW5/GW5*, I: Indica II type of *qSW5/GW5*, and N: Nipponbare type of *qSW5/GW5*

J: HJX74 type of *GW8*, B: Basmati type of *GW8*, and T: TN1 type of *GW8*

For LWR, significant differences were observed between alleles of five genes (*GS3*, *GS5*, *qSW5/GW5*, *GS6*, and *GW8*/*OsSPL16*) (Table 1). Of the 162 possible allele combinations, 22 were observed in a total of 189 germplasms and were named group RA–RV (Additional file 3). Of these, the average LWR was significantly different in groups containing single gene-specific allelic variation at one of two genes (*GS3* or *qSW5/GW5*), particularly when certain allele combinations of the other three genes were present (Table 2). For instance, the average LWR was significantly different between groups RN and RP, which varied only at *qSW5/GW5* gene.

Significant allelic differences for KGW were observed in four genes (*GW2*, *qSW5*/*GW5*, *GS6*, and *GW8*/*OsSPL16*) (Table 1). Of the 36 possible allele combinations, 14 were found in a total of 206 germplasms and were termed group KA–KN (Additional file 3). Of these, KGW was significantly different in groups containing single gene-specific allelic variation only at *GW8*/*OsSPL16* gene, particularly when certain allele combinations of the other three genes were present (Table 2). For instance, KGW was significantly different between KM and KN, which varied only at *GW8*/*OsSPL16* gene.

Our results, taken together, indicate that a certain type of allele combination plays an important role in regulation of rice grain size and yield, even in the presence of difference genetic backgrounds. The results also suggest that particular allele combinations have a strong influence on single gene-specific allelic variation for grain size and yield.

### Development of a Regression Equation Model

Allelic distribution data for the six genes examined in this study were used to develop a regression equation model. Allele data were first converted into dummy variables. Genes with significant allelic associations with grain size-related traits (GL, GW, LRW, and KGW) were used as independent variables (Table 1). Regression analysis was performed using these data. Parameter estimates, standard deviations, and, t values were calculated accordingly (Tables 3 and 4). These values indicated the contribution levels of each molecular marker to differences in grain size-related traits. For instance, the influence of allelic variation at *GW2* gene in GW and KGW was significantly analyzed by use of a dCAPS marker, GW2-*Sca*I. These values were used to develop a regression equation model for prediction of rice grain size.

**Table 3:** Regression equation model for prediction of rice grain size

			Grain length		Grain width		Grain length to width ratio		1000-grain weight	
Gene name	Primer name	Variable	Parameter estimate	*T value*	Parameter estimate	*T value*	Parameter estimate	*T value*	Parameter estimate	*T value*
*GS3*	*GS3-PstI*	*GS3-1*	-0.901±0.17	-5.24**	0.1169±0.06	1.84	-0.4724±0.08	-5.34**	-1.2166±0.94	-1.29
		*GS3-2*	-1.4351±0.17	-8.17**	0.229±0.06	3.52**	-0.7206±0.09	_-_7 97**	-1.9387±0.96	-2*
*GS5*	*GS5-TaqI, GS5-Sal*I	*GS5-1*	-	-	-0.0607±0.05	1.15	-0.0769±0.07	-1.05	-	
		*GS5-2*	-	-	-0.0632±0.07	-0.81	0.0764±0.1	0.7	-	
*GS6*	indel-GS6	*GS6*	0.0008±0.12	0.01	-0.1564±0.04	-3.28**	0.1532±0.06	2.3*	-2.4581±0.68	-3.59**
*GW2*	GW2-*Sca*I	*GW2*	-	-	0.4838±0.16	2.87**	-	-	20.9539±2.53	8.26**
*qSW5/GW5*	N1212del	*qSW5-1*	0.7148±0.15	4.53**	-0.4591±0.05	-7.89**	0.7302±0.08	8.99**	-	-
		*qSW5-2*	0.0862±0.18	0.46	-0.0188±0.07	-0.27	0.1054±0.09	1.08	-	-
*GW8/OsSPL16*	indel-GW8, seq-GW8	*GW8-1*	0.5161±0.15	3.32**	-0.1267±0.05	-2.21*	0.2907±0.07	3.64**	0.7881±0.78	1
		*GW8-2*	0.2797±0.17	1.61	-0.2075±0.06	-3.24**	0.2845±0.08	3.18**	-1.4489±0.92	-1.57
	intercept		8.7539±0.24	35.67**	3.2556±0.09	35.95**	2.7589±0.12	21.83**	29.4539±0.91	32.13**
	total R		0.4391		0.5913		0.6339		0.3286	

** and * indicate the significance at 1 % and 5 % level, respectively

A Dummy variable substitution;

*GS3*: A-allele (0,0), B-allele (1,0), and C-allele (0,1)

*GS5*: H94 type (0,0), Zhonghua 11 type (1,0), and Zhenshan 97 type (0,1)

*GS6*: Type I (0) and Type II/III (1)

*GW2*: FAZ1 allele (0) and WY3 allele (1)

*qSW5/GW5*: Indica II type (0,0), Kasalath type (1,0), and Nipponbare type (0,1)

GW8/OsSPL16: Basmati allele (0,0), HJX74 allele (1,0), and TN1 allele (0,1)

**Table 4:** Regression equation model for prediction of rice grain size

Regression equation models	
Grain length	8.7539+0.0008(GS6)+0.0094(GS5_1)+0.0133(GS5_2)+0.7148(qSW5_1)+0.0862(qSW5_2)-0.9010(GS3_1)-1.4351(GS3_2)+0.5161(GW8_1)+0.2797(GW8_2)
Grain width	3.2556-0.1564(GS6)+0.0607(GS5_1)-0.0632(GS5_2)+0.4838(GW2)-0.4591(qSW5_1)-0.0188(qSW5_2)+0.1169(GS3_1)+0.2290(GS3_2)- 0.1267(GW8_1)-0.2075(GW8_2)
Grain length to width ratio	2.7589+0.1532(GS6)-0.0769(GS5_1)+0.0764(GS5_2)+0.7302(qSW5_1)+0.1054(qSW5_2)-0.4724(GS3_1)-0.7206(GS3_2)+0.2907(GW8_1)+0.2845(GW8_2)
1000-grain weight	3.2556-0.1564(GS6)+0.0607(GS5_1)-0.0632(GS5_2)+0.4838(GW2)-0.4591(qSW5_1)-0.0188(qSW5_2)+0.1169(GS3_1)+0.2290(GS3_2)- 0.1267(GW8_1)-0.2075(GW8_2)

### Validation of a Regression Model

**Fig. 1:**
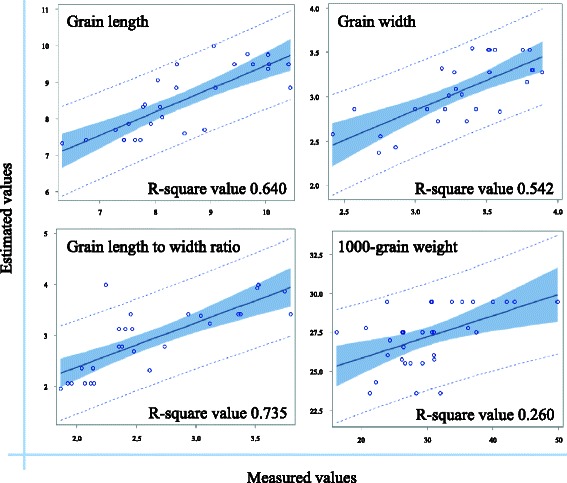
Validation of the regression equation model. The regression equation model was evaluated by comparing estimated values with actual measured values for four grain size-related traits (GL, GW, LWR, and KGW). The *x*-axis indicates measured values and the *y*-axis indicates estimated values. The dotted line indicates the 95 % prediction limits, the blue-shaded region indicates the 95 % confidence limits, and the solid line indicates the best prediction from the model

To evaluate the regression equation model, allele and grain size-related trait data were gathered from an additional 34 germplasms of diverse origin and grain size (Additional file 5). The estimated values of each grain size-related trait (GL, GW, LRW, or KGW) were calculated using the regression equation model, and these values were then compared with actual measurements. The model had substantial predictive power for three traits (GL, R^2^ = 0.640; GW, R^2^ = 0.542; and LWR, R^2^ = 0.735) (Fig. 1). However, predictive power for KGW was low (R^2^ = 0.260), suggesting that grain weight is possibly regulated in a complex manner compared to grain length and width.

## Discussion

### Discovery of an Additional Allele of *GS3* Gene

A causal C to A mutation in the second exon of the *GS3* gene is highly associated with GL in rice (Fan et al. 2009). This mutation, which creates a prematurely truncated GS3 protein, results in enhanced GL. Here, 110 of the 215 germplasms tested had A- (C165A mutation) or C-alleles (Table 1). These germplasms differed in GL according to their *GS3* allelic variation. The remaining 104 germplasms contained a novel allele (termed the B-allele) (Additional file 6), which had a 45-bp insertion in the first intron and did not exhibit the C165A mutation (Takano-Kai et al. 2013). Germplasms carrying the B-allele of *GS3* gene had an intermediate GL compared to the germplasms carrying the A- and C-alleles (Table 1). As noted above, enhanced GL in C165A varieties was attributed to the truncated GS3 protein (Mao et al. 2010). It has been reported that aberrant splicing can be generated by insertions in intron regions (Sironen et al. 2006). We propose that such an aberrant splicing event could have been generated by the 45-bp intronic insertion in the B-allele, and that this led to modified translation of GS3 protein. Comparisons of the B- and C-allele transcripts would verify such aberrant splicing. The presence of the novel *GS3* allele in a large proportion of the tested germplasms highlights its importance. The B-allele will enhance our understanding of the influence of *GS3* on grain size-related traits.

### The Influence of Allele Combinations from Six Genes on Rice Grain Size

A large number of genes are involved in regulation of rice grain size, and there are consequently thousands of possible allele combinations governing grain traits. Here, we examined the influence of allelic variation in six genes on grain size-related traits: *GS3*, *GS5*, *GS6*, *GW2*, *GW8*/*OsSPL16*, and *qSW5*/*GW5*. Allele data and grain parameters were used to develop and test a regression equation model for prediction of grain size. Our results showed that a relatively small number of allele combinations persisted in the diverse rice germplasm collection, and that these combinations were significantly associated with differences in grain size. We also noted that single gene-specific allelic variation played an important role in regulation of grain size in the presence of certain allele combinations. For example, GL was significantly different between allelic groups LA, LB, and LC, which had the same alleles for four of the five genes under consideration, but not between LD–LF or LJ–LL, which also differed at only one of the five genes (Additional file 4). These results suggest that particular allele combinations have substantial influences on rice grain size.

### Application of a Regression Equation Model for Prediction of Rice Grain Size

A regression equation model was generated using allele and trait data from 215 germplasms. The model was then tested against data from a further 34 diverse germplasms. R-square values indicative of correlation were obtained for three grain size-related traits (GL, 0.640; GW, 0.542; and LWR, 0.735), but correlation was low for KGW (R^2^ = 0.260) (Fig. 1). These values indicate the utility of our regression equation model for prediction of grain size in rice. Our data show that grain size is likely to be strongly influenced by combinations of certain alleles, and the regression equation model therefore provides a useful tool for rice molecular breeding. For example, rice grain size preferences vary between countries, and the regression equation model could be used to develop novel rice varieties with grain length and width within a desirable range. However, it is important to note that, although the R-square values seen in this study indicate some correlation, the current model is limited in its predictive ability as only six genes were examined. The model will be improved by inclusion of novel alleles as they are discovered (such as the B-allele of *GS3* discovered in this study) and by addition of data from more genes. In addition, the model developed here can be used alongside other models to refine breeding for other yield potential and grain quality traits. For example, several regression equation models have been developed to estimate rice eating quality (Lestari et al. 2009; Lestari et al. 2015).

## Conclusion

Allelic variation of six key genes involved in regulation of rice grain size was widely distributed in a collection of 215 germplasms, indicating that the collection was representative of genetic diversity in rice (*Oryza sativa* L.). Several germplasms in our collection that had similar grain traits (such as length and width) also shared allele combinations. These results suggest that rice grain size is likely determined by particular allele combinations of several genes involved in regulation of grain size. The results were used to develop a regression equation model that can be used for rice molecular breeding programs. Our data and regression model will be valuable for market-targeted rice breeding programs such as those aimed at different grain size preferences in different countries.

## Methods

### Plant Materials and Grain Size Measurements

A total of 215 rice germplasms that were developed in, or originated from, 28 rice-consuming countries were used in this study (Additional file 1). Rice plants were examined under natural field conditions in the experimental farm of Seoul National University, Suwon, Korea. Normal agricultural practice was followed for field management. Harvested rice grains were air-dried for 1 month prior to measurement. A total of ten fully filled grains were randomly chosen for each rice germplasm. The size of selected grains (GL, GW, and LWR) was measured using SmartGrain Version 1.2 software (Tanabata et al. 2012). The KGW of each germplasm was determined by measuring 200 fully filled grains and multiplying by five.

### Genomic DNA Extraction, PCR, and Sequencing

Genomic DNAs were extracted from young rice seedlings using a modified CTAB DNA extraction procedure (Murray and Thompson 1980). PCR was performed in a reaction volume of 20 μL containing 40 ng of genomic DNA, 0.2 μM of each primer, 200 μM of each dNTP, 10 mM Tris–Cl (pH 8.3), 50 mM KCl, 1.5 mM MgCl_2_, 0.01 % gelatin, and 0.5 U of Taq DNA polymerase. PCR amplifications were carried in a DNA Engine Tetrad 2 Peltier Thermal Cycler (Bio-Rad) using the following program: 5 min at 94 °C; 32 cycles of 45 s at 94 °C, 30 s at 47–65 °C, 20 s at 72 °C; and 10 min final extension at 72 °C (Additional file 7). Amplified PCR products were separated on a 1–3 % agarose gel to validate the expected fragment size.

### Statistical Analysis

All statistical analyses were performed using SAS statistical software (SAS Enterprise Guide 6.1 and SAS 9.4; http://www.sas.com/). ANOVA test (including Duncan’s test) was used to determine significant associations between the six selected genes and four grain size-related factors (GL, GW, LWR, and KGW). For multiple regression analysis, allelic variation of the six genes was converted to dummy variables. Allelic variation was scored in two ways, depending on the number of alleles: 1 (allele A) and 0 (allele B); or 00 (allele A), 10 (allele B), and 01 (allele C). The genes that showed significant associations with grain size-related traits were further verified examined using general linear model analysis (McCullagh 1984). Measured values were considered as dependent variables, while binary molecular marker data were considered as independent variables (Lestari et al. 2009). These data were used to develop a regression equation model for prediction of rice grain size.

## Additional files

Additional file 1:
**A list of germplasms.** (XLSX 62 kb)
Additional file 2:
**Relationships between grain size-related traits.** (XLSX 8 kb)
Additional file 3:
**Allele combination of genes showing significant associations with grain size-related traits.** (XLSX 17 kb)
Additional file 4:
**A single gene-specific allelic variation at** ***GS3.*** (XLSX 11 kb)
Additional file 5:
**A list of germplasms for evaluation of the regression equation model.** (XLSX 16 kb)
Additional file 6:
**Electrophoresis profiles of** ***GS3*** **analysis with the dCAPS marker.** PCR products were amplified using GS3-*PstI* and then digested with *Pst*I at 37 °C. Digested products were separated on a 2.5 % agarose gel. 1: undigested (A-allele, 512 bp), 2 + 4: digested (B-allele, 339 bp + 218 bp), and 3 + 4: digested (C-allele; 294 bp + 218 bp). (PDF 96 kb)
Additional file 7:
**A list of primers.** (XLSX 9 kb)

## Abbreviations

ANOVA: Analysis of Variance
dCAPS: Derived Cleaved Amplified Polymorphic Sequence
EMS: Ethylmethanesulfonate
FAZ1: Fengaizhan 1
FNP: Functional Nucleotide Polymorphism
GL: Grain Length
GW: Grain Weight
InDel: Insertion-deletion
KGW: 1000-grain Weight
LWR: Grain Length to Width Ratio
QTLs: Quantitative Trait Loci
SNP: Single Nucleotide Polymorphism
UTR: Untranslated Region
WY3: Wuyujing 3
