# Erratum to: ‘Influence of Multi-Gene Allele Combinations on Grain Size of Rice and Development of a Regression Equation Model to Predict Grain Parameters’

**DOI:** 10.1186/s12284-016-0128-z

**Published:** 2016-10-07

**Authors:** Chan-Mi Lee, Jonghwa Park, Backki Kim, Jeonghwan Seo, Gileung Lee, Su Jang, Hee-Jong Koh

**Affiliations:** Department of Plant Science, Plant Genomics and Breeding Institute, Research Institute for Agriculture and Life Sciences, Seoul National University, Seoul, 151-921 South Korea

## Erratum

Unfortunately, the original version of this article (Lee et al. 2015) contained an error. In Table 4 (Regression equation model for prediction of rice grain size), model equations for Grain width and fro 1000-weight were duplicated. Below is the corrected Table 4.

**Table 4:** Regression equation model for prediction of rice grain size

Regression equation model	
Grain length	8.7539 + 0.0008(GS6) + 0.0094(GS5_1) + 0.0133(GS5_2) + 0.7148(qSW5_1) + 0.0862(qSW5_2)-0.9010(GS3_1)-1.4351(GS3_2) + 0.5161(GW8_1) +0.2797(GW8_2)
Grain width	3.2556-0.1564(GS6) + 0.0607(GS5_1)-0.0632(GS5_2) + 0.4838(GW2)-0.4591(qSW5_1)-0.0188(qSW5_2) + 0.1169(GS3_1) + 0.2290(GS3_2)-0.1267(GW8_1) -0.2075(GW8_2)
Grain length to width ratio	2.7589 + 0.1532(GS6)-0.0769(GS5_1) + 0.0764(GS5_2) + 0.7302(qSW5_1) + 0.1054(qSW5_2)-0.4724(GS3_1)-.7206(GS3_2) + 0.2907(GW8_1) + 0.2845(GW8_2)
1000-grain weight	29.4539-2.4581(GS6) + 20.9539(GW2)-1.2166(GS3_1)-1.9387(GS3_2) + 0.7881(GW8_1)-1.4489(GW8_2)

## References

[CR1] Lee CM, Park J, Kim B, Seo J, Lee G, Jang S, Koh HJ (2015). Influence of Multi-Gene Allele Combinations on Grain Size of Rice and Development of a Regression Equation Model to Predict Grain Parameters. Rice.

