# Supplementary figures and images for: Influence of Multi-Gene Allele Combinations on Grain Size of Rice and Development of a Regression Equation Model to Predict Grain Parameters
Source: Rice (N Y). 2015 Oct 30;8:33. doi: 10.1186/s12284-015-0066-1 (PMC4627975; doi:10.1186/s12284-015-0066-1)

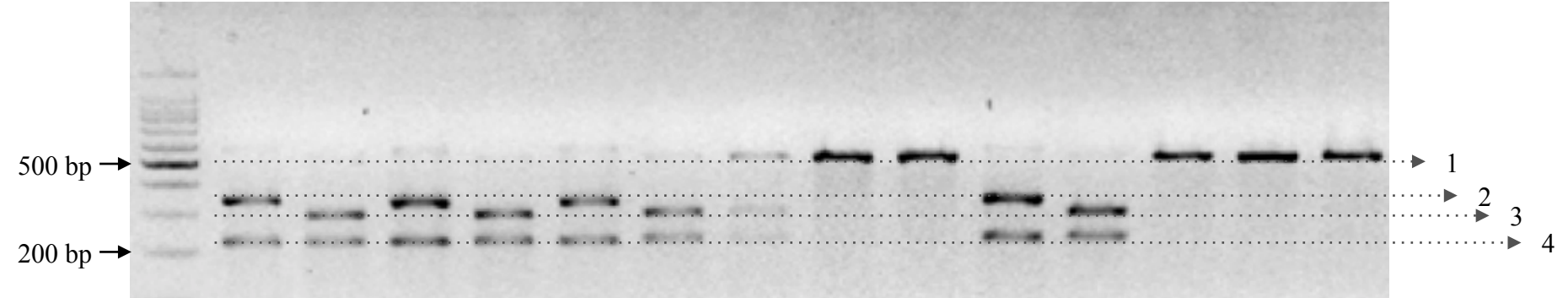

Supplement: Additional file 6: — Electrophoresis profiles of GS3 analysis with the dCAPS marker. PCR products were amplified using GS3-PstI and then digested with PstI at 37 °C. Digested products were separated on a 2.5 % agarose gel. 1: undigested (A-allele, 512 bp), 2 + 4: digested (B-allele, 339 bp + 218 bp), and 3 + 4: digested (C-allele; 294 bp + 218 bp). (PDF 96 kb) [file 12284_2015_66_MOESM6_ESM.pdf]
